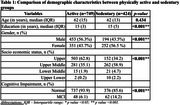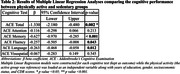# Assessing the impact of physical activity on cognitive function among urban elderly population: a cross‐sectional analysis

**DOI:** 10.1002/alz.091278

**Published:** 2025-01-03

**Authors:** Deva Kumar HS, Monisha Selva, Abhishek Mensegere Lingegodwa, Albert Stezin, Ajith Partha, Divya N Mallikarjun, Amitha C M, Rajitha Narayanasamy, Meenakshi Menon, Meghana R, Vindhya Vishwanath, Goutham Velavarajan, Palash K Malo, Prathima Arvind, Shafeeq K Shahul Hameed, Sunitha HS, Banashree Mondal, Deepashri Agrawal, Jonas S. Sundarakumar, Thomas Gregor Issac

**Affiliations:** ^1^ Centre for Brain Research, Indian Institute of Science, Bangalore, Karnataka India; ^2^ Centre for Brain Research, Bangalore India

## Abstract

**Background:**

Evidence from multiple converging sources has demonstrated the clear benefits of physical activity in promoting mental health and improving cognitive function. However, more than 54% of Indians do not engage in the recommended amount of physical activity. The present study aims to explore the association of physical activity with cognitive abilities among the elderly aging population in an urban Indian setting.

**Methods:**

The study utilized baseline data of 1250 till Dec 2023 from the ongoing Tata Longitudinal Study of Ageing cohort study. We categorized subjects into physically active (PA) and sedentary groups (SG)based on the time they spend doing different types of physical activity in a typical week using the Global Physical Activity Questionnaire (GPAQ). Cognitive assessments were performed using Addenbrooke’s Cognitive Examination III (ACE‐III). Multiple linear regression was performed where cog variables were loaded as dependent variables, physical activity was a predictor, and covariates used were education, gender, socioeconomic status, and clinical dementia rating scores.

**Result:**

The mean age SD) of the study sample was 62.6 (9.6) years. Among 1250 participants, 804 (64.3%) were physically active and 446 (35.7%) belonged to the sedentary group. The physically active group had significantly higher median years of education (14.9 vs 14.6; p = <0.001), more proportion of males (56.3% vs 43.5%, p < 0.001), upper socioeconomic class (62.8% vs 34.2%) and lesser people with mild cognitive impairment (6.1% vs 14.2, p <0.001) as shown in Table 1. Multiple linear regression analysis shows that after controlling for confounding variables, the physically active group performed better in ACE total (β = ‐1.330, p = 0.002), ACE memory (β = ‐0.627, p < 0.001), ACE fluency (β = ‐0.257, p = 0.043) and ACE language (β = ‐0.263, p = 0.012) when compared to the sedentary group as shown in Table 2. ACE attention and ACE visuospatial scores were not statistically significant between both groups.

**Conclusion:**

Our findings indicate that physically active participants had better global cognition and better performance in memory, executive functioning, and perceptual‐motor function. Promotion of physical activity should hence be implemented for enhancing cognitive abilities in elderly individuals.